# Prediction Models for Conversion From Mild Cognitive Impairment to Alzheimer’s Disease: A Systematic Review and Meta-Analysis

**DOI:** 10.3389/fnagi.2022.840386

**Published:** 2022-04-07

**Authors:** Yanru Chen, Xiaoling Qian, Yuanyuan Zhang, Wenli Su, Yanan Huang, Xinyu Wang, Xiaoli Chen, Enhan Zhao, Lin Han, Yuxia Ma

**Affiliations:** ^1^Evidence-Based Nursing, School of Nursing, Lanzhou University, Lanzhou, China; ^2^Department of Neurology, Second Hospital of Lanzhou University, Lanzhou, China; ^3^Department of Nursing, Gansu Provincial Hospital, Lanzhou, China; ^4^First School of Clinical Medicine, Lanzhou University, Lanzhou, China

**Keywords:** mild cognitive impairment, Alzheimer’s disease, dementia, prediction models, systematic review

## Abstract

**Background and Purpose:**

Alzheimer’s disease (AD) is a devastating neurodegenerative disorder with no cure, and available treatments are only able to postpone the progression of the disease. Mild cognitive impairment (MCI) is considered to be a transitional stage preceding AD. Therefore, prediction models for conversion from MCI to AD are desperately required. These will allow early treatment of patients with MCI before they develop AD. This study performed a systematic review and meta-analysis to summarize the reported risk prediction models and identify the most prevalent factors for conversion from MCI to AD.

**Methods:**

We systematically reviewed the studies from the databases of PubMed, CINAHL Plus, Web of Science, Embase, and Cochrane Library, which were searched through September 2021. Two reviewers independently identified eligible articles and extracted the data. We used the Critical Appraisal and Data Extraction for Systematic Reviews of Prediction Modeling Studies (CHARMS) checklist for the risk of bias assessment.

**Results:**

In total, 18 articles describing the prediction models for conversion from MCI to AD were identified. The dementia conversion rate of elderly patients with MCI ranged from 14.49 to 87%. Models in 12 studies were developed using the data from the Alzheimer’s Disease Neuroimaging Initiative (ADNI). C-index/area under the receiver operating characteristic curve (AUC) of development models were 0.67–0.98, and the validation models were 0.62–0.96. MRI, apolipoprotein E genotype 4 (APOE4), older age, Mini-Mental State Examination (MMSE) score, and Alzheimer’s Disease Assessment Scale cognitive (ADAS-cog) score were the most common and strongest predictors included in the models.

**Conclusion:**

In this systematic review, many prediction models have been developed and have good predictive performance, but the lack of external validation of models limited the extensive application in the general population. In clinical practice, it is recommended that medical professionals adopt a comprehensive forecasting method rather than a single predictive factor to screen patients with a high risk of MCI. Future research should pay attention to the improvement, calibration, and validation of existing models while considering new variables, new methods, and differences in risk profiles across populations.

## Introduction

Alzheimer’s disease (often shortened to “Alzheimer’s” or “AD”) is the most common type of dementia occurring in older people and is defined as an irreversible, progressive neurodegenerative disorder characterized by abnormal accumulation of amyloid plaques and neurofibrillary tangles in the brain, causing a decline in thinking, memory, language, personality changes, and certain changes in the brain, that gradually get worse over time, eventually leading to a loss of ability to perform the simplest daily tasks (Ballard et al., 2011). As one of the greatest healthcare challenges of the twenty-first century, caring for patients with AD presents a heavy emotional and financial burden for families and society (Silva et al., 2019; Tiwari et al., 2019). Currently, AD affects more than 35 million people in the world, and its incidence is estimated to triple by 2050. In the United States alone, approximately 5.3 million people have AD, of which 5.1 million are aged 65 years or older (Alzheimer’s Association, 2015). The global socioeconomic costs for dementia were US$957.56 billion in 2015 and will reach US$2.54 trillion by 2030 and US$9.12 trillion by 2050 (Jia et al., 2018). Since current drug therapies can only postpone the progression of the disease and cannot directly prevent the progression of AD, more hope has been placed on the early prediction of AD.

Mild cognitive impairment (MCI) is considered to be a transitional stage between normal aging and AD and is a potential target for predicting individuals at risk of developing AD (Mariani et al., 2007). It can be defined as the presence of a memory complaint, objective memory impairment abnormal for age with relatively preserved general cognition, and essentially intact activities of daily living (no dementia) (Petersen, 2004). The annual conversion rate from MCI to AD has been reported as 10–15%. Approximately 80% of MCI patients will have converted to AD (Tábuas-Pereira et al., 2016), although some patients with MCI remain stable or convert back to normal (Lovell, 2009). Therefore, the predicted risk of conversion from MCI to AD and the early identification of patients with MCI with high risk are desperately required. These will facilitate the early treatment of patients with MCI before they convert to AD.

A systematic review of dementia prediction models was published in 2019, which reviewed the predictive performance and common predictors of dementia prediction models, but did not carry out an in-depth analysis of the prediction models and predictors of people for conversion from MCI to AD (Hou et al., 2019). In previous research, strenuous efforts had been made on the classification and prediction of MCI and AD based on the clinical, genetic, proteomic data and also on the AD imaging biomarkers. However, the predictive performance and clinical applicability of the current model still need to be further verified.

The purpose of this systematic review was to summarize the reported risk prediction models and identify the most prevalent factors for conversion from MCI to AD, so as to provide a theoretical basis for the construction, application, and optimization of the risk prediction model of dementia in patients with MCI and the early intervention.

## Methods

### Search Strategy

**FIGURE 1:**
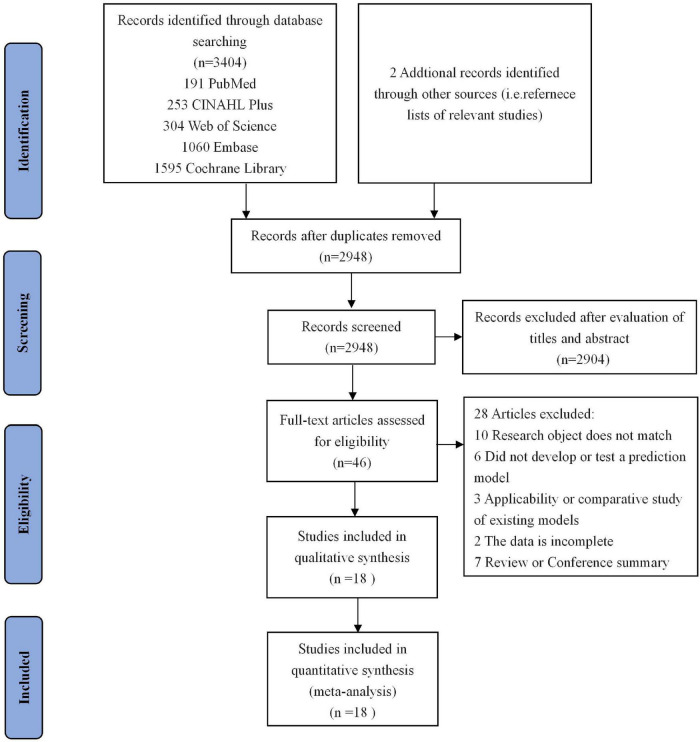
Flow chart of study selection. Showing the process by which relevant studies retrieved from the databases were assessed and selected or excluded.

We searched PubMed, CINAHL Plus, Web of Science, Cochrane Library, and Embase databases from the database inception through September 2021. The search strategies were performed through a combination of MeSH terms and free words. The following MeSH terms and free words were used: “cognitive dysfunctions,” “cognitive impairments,” “cognitive defect,” “mental disorders,” “dementia,” “amentias,” “Alzheimer’s disease,” “Alzheimer Syndrome,” “demention,” “prognostic model,” “prediction tool,” “prediction model,” “risk model,” and so on. Only articles published in English were considered for review. Additionally, the reference lists included in the identified articles were manually searched to identify additional relevant publications. The precise search strategies are shown in Figure 1.

### Eligibility Criteria

Two researchers (Chen and Su) did the literature search and extracted the data independently. Discrepancies were resolved by a third researcher (Ma).

The inclusion criteria of a study in the systematic review were as follows: (1) patients diagnosed with MCI; (2) the content of the study is the risk prediction model for conversion from MCI to AD; (3) the model has been built after internal or external validation; (4) study type is either a cohort study or a case-control study. The exclusion criteria were as follows: (1) Review articles; (2) reports; (3) commentary, abstracts, and presentations; (4) disease-specific dementia; (5) the process or method of model building is not described; (6) cannot access the original text or incomplete data; (7) republished literature.

### Data Extraction

Key information of the included studies was extracted by two researchers working independently using a data extraction form. From each study, we abstracted the following: first author, publication year, country, research object and location, dementia diagnostic criteria, modeling method, model verification method, predictive factors included in the model, area under the receiver operating characteristic curve (AUC), and model prediction performance.

### Quality Evaluation

The quality of the included studies was evaluated independently by two investigators, using the CHARMS (Moons et al., 2014). Any disagreement regarding the quality of studies was resolved by a third investigator. It included bias risk and applicability and was assessed from 11 items; the main items of which included source of data, participants, outcome(s) to be predicted, candidate predictors, sample size, missing data, model development, model performance, model evaluation, results, interpretation, and discussion.

### Statistical Analysis

We used descriptive analysis methods to sort out and summarize the basic characteristics of the included studies and models, development methods, verification methods, and prediction factors in the model. Stata 15.1 software was used for the meta-analysis of the predictive value of the predictors in the model. First, a *Q*-test was used to verify whether there was heterogeneity among the included models. The degree of heterogeneity was assessed using the *I*^2^ statistic, with the *I*^2^ values of 25, 50, and 75% being considered to indicate low, moderate, and high heterogeneity, respectively. If *I*^2^ > 50%, heterogeneity was considered larger, and the random effects model was used for analysis, otherwise the fixed effects model was used. The count data were represented by odds ratio (OR) and 95%CI, while the measurement data were represented by weighted mean difference (WMD) and 95%CI.

## Results

### Literature Search

Figure 1 shows the results of literature searching and selection. A total of 3,404 related articles were retrieved from the database, and 2,948 articles remained after deduplication. By reading the titles and abstracts, 2,904 articles were excluded from the study, and 28 articles were excluded from the study for reasons, such as failure to construct a risk prediction model, for the conversion from MCI to AD and repeated publication. In addition, two references were supplemented by consulting the included references. Ultimately, a total of 18 studies met the inclusion criteria and were utilized for the meta-analysis.

### Characteristics of Eligible Studies

A total of 18 studies were included in this study. Notably, 12 of the studies’ data were conducted using the data from the ADNI dataset. Of the 18 studies we included, 14 were retrospective cohort studies and four were prospective cohort studies. Among the diagnostic criteria for predicting the outcome of AD, five studies (Fleisher et al., 2008; Hansson et al., 2009; Korolev et al., 2016; Handels et al., 2017; Jang et al., 2018) used NINCDS-ADRDA diagnostic criteria, while other studies used other criteria, such as Diagnostic and Statistical Manual of Mental Disorders (DSM), Mini-Mental State Examination (MMSE), Alzheimer’s Disease Assessment Scale cognitive (ADAS-cog) scale, and Clinical diagnostic assessments. Shigemizu’s study (Shigemizu et al., 2020) uses the National Institute on Aging-Alzheimer’s Association workgroups on diagnostic guidelines for AD (NIA) as the diagnosis of outcome indicators. The follow-up duration of the studies ranged from 0.5 to 7 years, and the dementia conversion rate of elderly patients with MCI ranged from 14.49 to 87%. The basic characteristics of the included studies are shown in Table 1.

**TABLE 1:** Primary characteristics of the prediction model included in the review (*n* = 18).

Source	Country	Type of study	Source of data	Subject	AD assessment tool	Follow-up time	Incidence of AD
Basaia et al. (2019)	United States/Italy	Retrospective cohort study	ADNI/Milan dataset	AD, MCI and healthy controls	Clinical assessment	3 years	33%/54%
Ding et al. (2017)	United States	Retrospective cohort study	ADNI dataset	AD, MCI and healthy controls	NR	1 years	14.49%
Fleisher et al. (2008)	United States	Retrospective cohort study	ADCS MCI treatment trial	aMCI	NINCDS-ADRDA	3 years	41.09%
Han et al. (2017)	United States	Retrospective cohort study	ADNI dataset	MCI	NR	NR	60.18%
Handels et al. (2017)	Multicountry, multicenter	Retrospective cohort study	DESCRIPA multicenter study; LEARN multicenter study; Ljubljana University Medical Center and Karolinska University Hospital Huddinge memory clinic	MCI	DSM-IV-TR and NINCDS-ADRDA	2 years	87%
Hansson et al. (2009)	Sweden	Prospective cohort study	Malm¨o University Hospital	MCI and healthy controls	DSM-IIIR and NINCDS-ADRDA	4∼6 years	41%
Huang et al. (2019)	United States	Retrospective cohort study	ADNI dataset	MCI	CDR scores	At least 0.5 years	26.21%
Jang et al. (2018)	South Korea	Prospective cohort study	Memory Disorder Clinic in Samsung Medical Center, Clinical Research Center for Dementia of South Korea	aMCI	DSM-IV and NINCDS-ADRDA	3 years	61.5%
Jang et al. (2019)	United States	Retrospective cohort study	ADNI dataset	Aβ + MCI	Clinical diagnostic assessments	3 years	41.94%
Kauppi et al. (2018)	United States	Retrospective cohort study	ADNI dataset	MCI	NR	3 years	54.17%
Korolev et al. (2016)	United States	Retrospective cohort study	ADNI dataset	MCI	NINCDS-ADRDA	3 years	53.67%
Lee et al. (2019)	United States	Retrospective cohort study	ADNI dataset	AD, MCI and healthy controls	NR	2 years	35.49%%
Li et al. (2019)	United States	Retrospective cohort study	ADNI dataset	MCI	ADAS-cog, RAVLT, FAQ and MMSE	1 years	38.32%
Pereira et al. (2017)	Portugal	Prospective cohort study	CCC	MCI	DSM-IV	5 years	36.00%
Prins et al. (2013)	Netherlands	Prospective cohort study	Gal-Int-11	MCI	CDR scores	2 years	19.00%
Sabuncu (2013)	United States	Retrospective cohort study	ADNI dataset	MCI	NR	4.5 years	42.24%
Shigemizu et al. (2020)	Japan	Retrospective cohort study	NCGG	MCI	NIA	0.5∼7 years	42.10%
Sörensen et al. (2019)	Germany	Retrospective cohort study	ADNI dataset	MCI	NR	3.8∼4 years	33.27%

*ADNI, Alzheimer’s Disease Neuroimaging Initiative; AD, Alzheimer’s disease; MCI, mild cognitive impairment; ADCS, Alzheimer’s Disease Cooperative Study; aMCI, amnestic mild cognitive impairment; NINCDS-ADRDA: National Institute of Neurological and Communicative Disorders-Stroke/Alzheimer’s Disease and Related Disorders Association; DSM: Psychiatry Diagnostic & Statistical Manual of Mental Disorders; Aβ + MCI, Aβ positive (+) mild cognitive impairment; ADAS-cog scale: Alzheimer’s Disease Assessment Scale cognitive subscale; RAVLT, Rey Auditory Verbal Learning Test; FAQ, Functional Assessment Questionnaire; MMSE: mini-mental state examination score; CCC, the Cognitive Complaints Cohort; Gal-Int-11, the Galantamine-International-11 trial; CDR scores: cognitive dementia rating scores; NCGG, the National Center for Geriatrics and Gerontology; NIA: the criteria of the National Institute on Aging Alzheimer’s Association workgroups.*

### Evaluation of Methodology Quality in Eligible Studies

**FIGURE 2:**
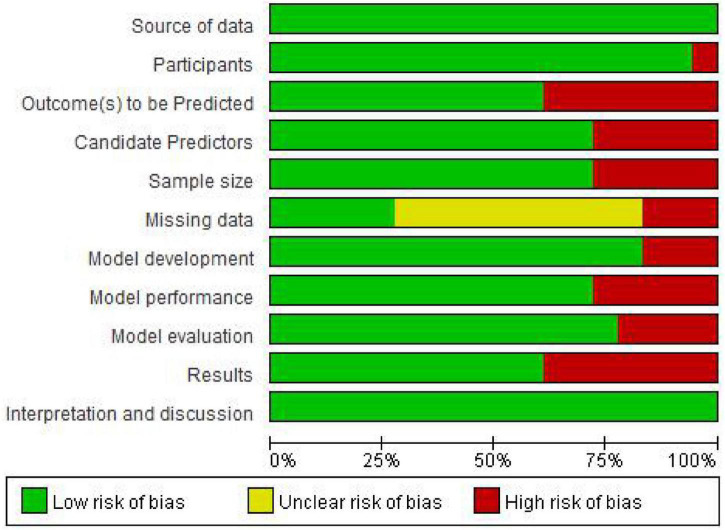
Risk of bias assessment for included studies.

All the articles included in this study were prospective or retrospective cohort studies, and the inclusion and exclusion criteria of the subjects were presented. Of note, eight studies (Prins et al., 2013; Korolev et al., 2016; Ding et al., 2017; Handels et al., 2017; Pereira et al., 2017; Jang et al., 2019; Lee et al., 2019; Shigemizu et al., 2020) reported missing data, and five of them processed missing data by mean interpolation (Pereira et al., 2017), excluded missing data cases (Korolev et al., 2016; Jang et al., 2019; Shigemizu et al., 2020), and multiple imputation (Handels et al., 2017); 11 studies (Fleisher et al., 2008; Hansson et al., 2009; Prins et al., 2013; Korolev et al., 2016; Handels et al., 2017; Pereira et al., 2017; Jang et al., 2018; Kauppi et al., 2018; Basaia et al., 2019; Huang et al., 2019; Sörensen et al., 2019) clearly defined predictive outcome indicators, and five studies (Fleisher et al., 2008; Hansson et al., 2009; Prins et al., 2013; Handels et al., 2017; Basaia et al., 2019) used blind methods for researchers assessing predictors of outcomes; 13 articles used single factor analysis, multiple factor analysis, and literature review for selected predictors. In terms of model verification, 12 studies (Fleisher et al., 2008; Sabuncu, 2013; Korolev et al., 2016; Han et al., 2017; Handels et al., 2017; Kauppi et al., 2018; Basaia et al., 2019; Huang et al., 2019; Jang et al., 2019; Lee et al., 2019; Sörensen et al., 2019; Shigemizu et al., 2020) only carried out internal verification, such as bootstrap method and cross-validation (CV), but did not conduct external verification. Only two studies (Pereira et al., 2017; Jang et al., 2018) assessed the method using internal and external model verification. In addition, 11 articles (Fleisher et al., 2008; Hansson et al., 2009; Prins et al., 2013; Korolev et al., 2016; Han et al., 2017; Handels et al., 2017; Jang et al., 2018; Kauppi et al., 2018; Jang et al., 2019; Sörensen et al., 2019; Shigemizu et al., 2020) presented complete regression equations, neural networks, nomograms, or Bayesian network models. The risk of bias assessment for included studies is shown in Figure 2.

### Basic Characteristics, Modeling, and Verification Methods

A total of 47 predictive models for the conversion from MCI to dementia were reported in the 18 articles included. In each study, we selected a model validated by the author or with the best performance in C-index/AUC for analysis and verification and statistical analysis. The first predictive model was published in 2008 (Fleisher et al., 2008), the latest in 2020 (Shigemizu et al., 2020), and 14 in the last 5 years. Models in 12 studies were developed using the data from the ADNI dataset. The sample size of method development in the studies is 98–1,327. Cox regression (*n* = 7), nomogram (*n* = 3), and neural networks (*n* = 3) are the most widely used modeling methods. Other modeling methods, such as Logistic regression (*n* = 2), support vector machine (SVM; *n* = 1), probabilistic multiple kernel learning (*n* = 1), Bayesian algorithm (*n* = 1), and supervised learning, approach based on time windows (*n* = 1). The sample size of method validation is 62–865, with eight studies using CV (Fleisher et al., 2008; Sabuncu, 2013; Korolev et al., 2016; Han et al., 2017; Kauppi et al., 2018; Basaia et al., 2019; Huang et al., 2019; Lee et al., 2019), three studies (Jang et al., 2018, 2019; Shigemizu et al., 2020) using a mix of CV and bootstrap validation, and only two studies (Pereira et al., 2017; Jang et al., 2018) using internal and external validation. Notably, nine of them reported the AUC value of the development model of 0.67–0.98, and 10 of them reported the AUC value of the validation model of 0.62–0.96.

Among the predictors included in the study models, a maximum of 10 predictors and a minimum of one predictor were reported. MRI (*n* = 12), apolipoprotein E genotype 4 (APOE4) (*n* = 10), older age (*n* = 7), female gender (*n* = 6), lower MMSE (*n* = 5), ADAS-cog scores (*n* = 5), cerebrospinal fluid (CSF) biomarkers (*n* = 5), and Functional Assessment Questionnaire (FAQ) scores (*n* = 4) were the most common predictors included in the models. Other than that, some other predictive factors, such as Fl8-fludeoxyglucose PET (FDG-PET; *n* = 3), education (*n* = 2), Rey Auditory Verbal Learning Test (RAVLT) scores (*n* = 2), and delayed recall test (*n* = 2), can also predict the conversion from MCI to dementia. In short, all predictors of models can be grouped into the following categories: demographic data (e.g., age, gender, education, and APOE ε4), cognitive scores (e.g., MMSE, ADAS-cog, FAQ, and RAVLT), fluid biomarkers (e.g., CSF: amyloid-β 1–42 (Aβ1–42), t-tau, and p-tau), and imaging biomarkers (e.g., MRI and FDG-PET). The modeling, verification methods, and predictors for included studies are shown in Table 2.

**TABLE 2:** Modeling, verification methods, and predictive factors for included studies (*n* = 18).

Source	Model development	Model validation	Risk factors in final model	Sample size		AUC		Accuracy	Sensitivity	Specificity
				Development	Validation	Development	Validation			
Basaia et al. (2019)	CNN	10-fold cross-validation	Brain structural MRI scan	1,327	147	NR	NR	74.90%	75.80%	74.10%
Ding et al. (2017)	SVM	NR	MRI (cortical thickness), FDG-PET (cerebellum and the whole white matter)	214	NR	0.67	NR	72.78%	46.67%	66.05%
Fleisher et al. (2008)	LR	10-fold cross-validation	APOE4 status, MRI (ventricular volumes and hippocampal volumes), ADAS-cog, NYU Delayed paragraph recall, and 10-word Delayed recall	129	129	NR	0.89	78.80%	NR	NR
Han et al. (2017)	CR	10-fold cross-validation	APOE ε4 allele, Neurological disorder (other than AD), CDR-SB, ADAS-cog 13, MMSE, FAQ	658	74	NR	0.84	77%	NR	NR
Handels et al. (2017)	LR	Bootstrap validation	Female gender, MMSE, MTA scores on MRI and CSF biomarkers (Aβ1–42, t-tau and p-tau)	250	250	NR	0.85	NR	NR	NR
Hansson et al. (2009)	CR	NR	Older age, female gender, APOE ε4 carrier, rCBF and CSF biomarkers (t-tau, p-tau and Aβ1–42)	167	NR	0.78	NR	77.2%	NR	NR
Huang et al. (2019)	Nomogram	10-fold cross-validation	Radiomics signature (MRI (cortical features)), FAQ scores and Aβ1–42 CSF concentrations	191	99	0.98	0.96	NR	NR	NR
Jang et al. (2018)	Nomogram	Bootstrapping, 10-fold cross-validation + External validation	Older age, APOE4 and neuropsychological features (modality, severity, and multiplicity)	167	75	0.80	0.75,0.82	NR	NR	NR
Jang et al. (2019)	Nomogram	Bootstrapping, 10-fold cross-validation	APOE4, MCI stage, MRI (hippocampal volume), FDG-PET SUVR, CSF (t-tau and P-tau)	124	62	0.93	0.91	NR	NR	NR
Kauppi et al. (2018)	CR	Cross-validated	APOE ε4 alleles, MMSE, MRI (brain atrophy score), and PHS	336	336	0.84	NR	78.9%	79.9%	77.8%
Korolev et al. (2016)	pMKL	10-fold cross-validation	Cognitive scores (ADAS-Cog and RAVLT), functional assessments (FAQ) and MRI measures (volume/cortical thickness of left hippocampus, middle temporal gyrus, and inferior parietal cortex)	259	259	NR	0.87	79.9%	83.4%	76.4%
Lee et al. (2019)	RNN	5-fold cross-validation	Cognitive score (executive functioning and memory), MRI (hippocampal volume and entorhinal cortical thickness), CSF biomarker (Aβ1–42, t-tau and p-tau), demographic data (age, gender, education, and APOE ε4)	865	865	NR	0.86	81%	84%	80%
Li et al. (2019)	RNN, CR	NR	Demographic data (age, gender, education, and APOE ε4), hippocampal MRI measures, Cognitive measures (ADAS-Cog13, RAVLT immediate, RAVLT learning, FAQ, and MMSE)	822	439	0.90	NR	NR	NR	NR
Pereira et al. (2017)	A supervised learning approach based on time windows	5-fold cross-validation and External validation	Neuropsychological data	719	604,115	0.88	0.76	NR	0.88	0.71
Prins et al. (2013)	CR	NR	Age, gender, MTA scores on MRI, ADAS-cog/MCI and Delayed recall test	426	NR	NR	NR	NR	NR	NR
Sabuncu (2013)	BN	5-fold cross-validation	Structural MRI (cortical thickness)	393	315	NR	0.62	NR	NR	NR
Shigemizu et al. (2020)	CR	3-fold cross-validation, Bootstrap resampling	24 miR-eQTLs, older age, gender, and APOE ε4	98	99	0.72	0.70	NR	NR	NR
Sörensen et al. (2019)	CR	Proportional hazard assumption	APOE ε4 alleles, MMSE, FDG-PET	272	272	NR	NR	NR	NR	NR

*CNN, Convolutional neural networks; AUC, area under the receiver operating characteristic curve; SVM, Support Vector Machine; FDG-PET, Fl8-fludeoxyglucose (FDG) positron emission tomography (PET); LR, Logistic regression; APOE, apolipoprotein E genotype; ADAS-cog, Alzheimer’s Disease Assessment Scale–Cognitive Subscale; CR, COX regression; CDR-SB, cognitive dementia rating scale sum of boxes; MMSE, mini-mental state examination score; FAQ, Functional Assessment Questionnaire; MTA, medial temporal lobe atrophy; CSF, cerebrospinal fluid; Aβ1–42, amyloid-β 1–42; t-tau, total tau; P-tau, phosphorylated tau; rCBF, regional cerebral blood flow; PHS, polygenic hazard score; pMKL, probabilistic multiple kernel learning; RNN, recurrent neural network; RAVLT, Rey Auditory Verbal Learning Test; BN, Bayesian algorithm; miR-eQTLs, microRNA expression quantitative trait loci.*

### Meta-Analysis for Predictive Factors

In this review, we analyzed the predictive value of the eight predictors of MRI, APOE4, age, gender, MMSE score, ADAS-cog, FAQ score, and FDG-PET with the highest frequency of entering the predictive model, on the predictive value of the risk of AD in patients from MCI. The results showed that the five influencing factors, such as MRI (*OR* = 1.419, 95%CI: 1.176–1.712, *P* = 0.000), APOE4 (*OR* = 1.877, 95%CI: 1.552–2.271, *P* = 0.000), older age (WMD = 1.073, 95%CI: 1.010–1.140, *P* = 0.023), MMSE score (WMD = 0.877, 95%CI: 0.573–1.182, *P* = 0.000), and ADAS-cog score (WMD = 4.211, 95%CI: 3.488–4.934, *P* = 0.000), were statistically significant (*P* < 0.05). Among them, older age (*I*^2^ = 75.5%) and MMSE score (*I*^2^ = 68.5%) showed large heterogeneity (*I*^2^ > 50%). The results are shown in Table 3.

**TABLE 3:** Results of meta-analysis for predictive factors.

Predictors	Number of studies	Estimation of combined				Heterogeneity test	
		*OR*/*WMD*	95%*CI*	*Z*	*P*	*I*^2^(%)	*P*
MRI	12	1.419	1.176∼1.712	3.65	0.000	0.0%	0.557
APOE4	10	1.877	1.552∼2.271	6.48	0.000	38.4%	0.112
Age	7	1.073*	1.010∼1.140	2.28	0.023	75.5%	0.003
Gender	6	1.235	0.960∼1.588	1.64	0.101	50.0%	0.075
MMSE	5	0.877*	0.573∼1.182	5.65	0.000	68.5%	0.042
ADAS-cog	5	4.211*	3.488∼ 4.934	11.41	0.000	25.6%	0.246
FAQ score	4	7.646*	-0.919∼6.210	1.75	0.080	98.7%	0.000
FDG-PET	3	0.748	0.280∼1.995	0.58	0.561	93.6%	0.000

*WMD.

*OR, odds ratio; WMD, Weighted Mean Difference; CI, confidence interval; MRI, magnetic resonance imaging; APOE, apolipoprotein E genotype; MMSE, Mini–Mental State Examination; ADAS-cog, Alzheimer’s Disease Assessment Scale–Cognitive Subscale; FAQ, Functional Assessment Questionnaire; FDG-PET, Fl8-fludeoxyglucose (FDG) positron emission tomography (PET).*

## Discussion

We conducted a systematic review of prediction models for conversion from MCI to AD and a meta-analysis of the performance of prediction factors. The 18 studies included in this systematic review are carefully designed in the process of model development and evaluation. The included studies are both prospective cohort studies and retrospective cohort studies, and they all define the source and inclusion criteria of the study objects, which effectively reduce the selection bias. Among the 47 predictive models reported, the AUC of 40 models in the model population were all > 0.7, indicating that the constructed model can accurately identify the risk of dementia with MCI. However, there are some shortcomings in the model construction process.

For example, of the studies we analyzed, 12 were based on the ADNI, with fewer studies from other independent cohorts, which will limit the generalizability of those models. During the validation of the model, most studies only use CV or bootstrap internal validation, only two studies (Pereira et al., 2017; Jang et al., 2018) used external validation, while it is well known that external validation is critical in assessing a model’s capability and applicability (Hou et al., 2019). In addition, the candidate refers to the predictors chosen to be studied for their predictive performance and is not restricted to those included in the multivariable analysis (Moons et al., 2012). The number of candidate predictors analyzed in the primary studies is highly important. However, a few studies (Fleisher et al., 2008; Sabuncu, 2013; Ding et al., 2017; Pereira et al., 2017; Basaia et al., 2019) have not reported the number and process of analyzing candidate predictors before developing models, which may lead to the overfitting of final prediction model (Moons et al., 2014). In future studies, it is recommended to conduct a multicountry, multicenter prospective cohort study to explore more generalized and applicable predictive tools and factors. At the same time, researchers should pay attention to improvement, calibration, and validation of the models and also to the screening of candidate prediction factors to improve the reliability of research results.

### Demographic Data (Apolipoprotein E Genotype 4 ε4, Age, and Gender)

The results of this study showed that APOE4 was identified as one of the strongest predictors of the transition from MCI to dementia, consistent with previous studies (Lambert et al., 2013; Kunkle et al., 2019). In the meta-analysis of prediction factors, APOE4 also showed high homogeneity and prediction performance. In 1993, APOE4, as an important risk marker of AD, was first reported by Corder et al. (1993) and has been validated in major cohorts around the globe in recent years (Rasmussen et al., 2015; Livingston et al., 2020). Previous studies have shown that APOE4 has several effects on AD. First, APOE4 interferes with Aβ clearance from the brain and is also processed into neurotoxic fragments (Mahley and Huang, 2012). Furthermore, APOE4 causes the disinhibition of the cyclophilin A signaling mechanism in the pericytes of the brain blood vessels, leading to a degeneration of these vessels, leakage of the blood-brain barrier, and tau-induced neurodegeneration and atrophy (Bell et al., 2012; Serrano-Pozo et al., 2021). However, no therapies directed at APOE are currently available. Although several therapeutic approaches have been successful in mouse models expressing human APOE alleles, including increasing or decreasing APOE levels, enhancing its lipidation, blocking the interactions between APOE and Aβ peptide, and genetically switching APOE4 to APOE3 or APOE2 isoforms, translation to human clinical trials has proven challenging (Serrano-Pozo et al., 2021).

Older age was also strongly predictive for patients with MCI to AD. The reason may be that aging acts through various biological mechanisms at the cellular or tissue level which lead to multisystem loss of reserve and function and affect the health of the body’s blood vessels (Fabbri et al., 2015). Besides, amyloid and tau pathologies as well as brain atrophy increase with age (Vemuri et al., 2017). Of all the models we analyzed, six used female gender as a predictor, but the meta-analysis in this review found that the heterogeneity among the analyzed studies was large and not statistically significant, which is not consistent with the results of previous studies (Vermunt et al., 2019; Yoo et al., 2020). It may be related to the population selection (e.g., the sample size for the development and validation of the model in Shigemizu et al., 2020, study was less than 100) and the follow-up time (e.g., Li et al., 2019, study was only followed for 1 year) of the included models, which needs to be further verified in future research. It is suggested that future prediction models should be further validated by increasing sample size or longer follow-up time.

### Cognitive Scores (Mini-Mental State Examination, Alzheimer’s Disease Assessment Scale Cognitive, and Functional Assessment Questionnaire)

Notably, 28% of models we selected considered MMSE as a high factor for AD. Other cognitive screening scales, such as ADAS-cog score and FAQ score, also show good prediction performance. This is consistent with the findings of McEvoy et al. (2009). But Mitchell (2015) pointed out that the MMSE when used alone is not a good tool for prediction of future decline in people with MCI. A combination of predictors would be more accurate in predicting progression from MCI to dementia.

Both FAQ and ADAS-cog are the frequently used indices of cognitive decline in AD. In recent years, they have also been seen as strong predictors of the conversion from MCI to AD. In this review, five studies selected ADAS-cog score as a predictor, and all had good predictive performance; it suggests that baseline impairment in multiple cognitive domains is predictive of future progression to dementia (Korolev et al., 2016). Patients with MCI with both memory and non-memory deficits have a greater risk of progression to AD than those with isolated memory deficits (Bozoki et al., 2001). Impairment in multiple cognitive domains, as measured by the performance on the ADAS-cog, can be viewed as reflecting a more advanced MCI stage. The selection of FAQ scores as predictors for conversion from MCI to AD indicates that a subtle but reliable impairment in functional status precedes the development of overt dementia in patients with MCI (Korolev et al., 2016). In addition, longitudinal glucose metabolism decline was associated with concurrent ADAS-cog and FAQ decline, which has a value in predicting the future cognitive decline of patients with MCI (Landau et al., 2011). In general, the cognitive scores of MMSE, ADAS-cog, and FAQ are cost-effective. Neuropsychological test performance is relatively easy to gather, compared to the more expensive MRI scan, PET scan, and CSF biomarkers which require invasive lumbar puncture.

### Imaging Biomarkers (Magnetic Resonance Imaging and Fl8-Fludeoxyglucose Positron Emission Tomography)

The MRI is one of the most widely studied imaging techniques because it is completely non-invasive, highly available, and inexpensive compared to PET and has an excellent contrast between different soft tissues (Johnson et al., 2012). The results of this review showed that MRI was identified as the strongest predictor of the transition from MCI to AD. In our review, 67% of models we selected considered MRI as a high factor for conversion from MCI to AD, and all had a good predictive performance. The AUCs ranged from 0.62 to 0.98. In general, models that combined, such as demographic data, cognitive scores, fluid biomarkers, and other clinical markers, have better predictive performance than a single MRI model. The results of our study showed that many MRI markers, such as the whole brain, hippocampal, entorhinal cortex atrophy, and medial temporal lobe atrophy (MTA) on MRI score, have significant predictive value. However, the systematic studies of advantages/disadvantages of various MRI markers have been limited so far, and the existing studies do not allow to make definite conclusions. There is still no preferred MRI representation for AD-conversion prediction (Gómez-Sancho et al., 2018).

Previous research has shown that FDG-PET is also one of the effective predictors for predicting the conversion from MCI to AD. However, since there were only three cases of meta-analysis in this review, and the model sample size of some studies was small, the results showed a large heterogeneity, which needs to be further verified in future research. In addition, FDG-PET is relatively expensive and, similar to all PET techniques, has more limited availability. It requires intravenous access and involves exposure to radioactivity. Brain FDG retention is a non-specific indicator of metabolism that can be deranged for a variety of reasons (e.g., ischemia or inflammation) and may, in certain individuals, be irrelevant or only indirectly related to any AD-related process.

## Conclusion

In this systematic review, many prediction models have been developed and have good predictive performance, but the lack of external validation of models limited the extensive application in the general population. In addition, 67% of models were based on the ADNI dataset, with fewer studies from other independent cohorts, which will limit the generalizability of those models. MRI, APOE4, older age, MMSE score, and ADAS-cog score were the most common and strongest predictors included in the models. However, a few of the predictors are still highly heterogeneous. In clinical practice, it is recommended that medical professionals adopt a comprehensive forecasting method rather than a single predictive factor to screen patients with a high risk of MCI. Furthermore, future research should pay attention to improvement, calibration, and validation of existing models while considering new variables, new methods, and differences in risk profiles across populations.

## Data Availability Statement

The original contributions presented in the study are included in the article/supplementary material, further inquiries can be directed to the corresponding author/s.

## Author Contributions

YC, XQ, and YM contributed to the study design. WS, YH, XW, and EZ contributed to the data collection. YZ, YC, and XC performed statistical analyses and interpretation of results. YC, XQ, and LH drafted the manuscript and edited the language. All authors participated in the critical revisions and approved the final version of the manuscript.

## Conflict of Interest

The authors declare that the research was conducted in the absence of any commercial or financial relationships that could be construed as a potential conflict of interest.

## Publisher’s Note

All claims expressed in this article are solely those of the authors and do not necessarily represent those of their affiliated organizations, or those of the publisher, the editors and the reviewers. Any product that may be evaluated in this article, or claim that may be made by its manufacturer, is not guaranteed or endorsed by the publisher.
